# Enhancing the control of doubly fed induction generators using artificial neural networks in the presence of real wind profiles

**DOI:** 10.1371/journal.pone.0300527

**Published:** 2024-04-17

**Authors:** Chaimae Dardabi, Abdelouahed Djebli, Hamid Chojaa, Hadoun Aziz, Abderrahman Mouradi, Mahmoud A. Mossa, Almoataz Y. Abdelaziz, Thamer A. H. Alghamdi

**Affiliations:** 1 Energetic Laboratory, Department of physics, Faculty of science Tetouan, Abdelmalek Essaadi University, Tetouan, Morocco; 2 Industrial Technologies and Services Laboratory, Higher School of Technology, Sidi Mohamed Ben Abdellah University, Fez, Morocco; 3 Energy, Materials and Computing Physics Research Group, ENS, Abdelmalek Essaadi University, Tetouan, Morocco; 4 Electrical Engineering Department, Faculty of Engineering, Minia University, Minia, Egypt; 5 Faculty of Engineering and Technology, Future University in Egypt, Cairo, Egypt; 6 Wolfson Centre for Magnetics, School of Engineering, Cardiff University, Cardiff, United Kingdom; 7 Electrical Engineering Department, Faculty of Engineering, A-Baha University, Al-Baha, Saudi Arabia; Huazhong University of Science and Technology, CHINA

## Abstract

This study tackles the complex task of integrating wind energy systems into the electric grid, facing challenges such as power oscillations and unreliable energy generation due to fluctuating wind speeds. Focused on wind energy conversion systems, particularly those utilizing double-fed induction generators (DFIGs), the research introduces a novel approach to enhance Direct Power Control (DPC) effectiveness. Traditional DPC, while simple, encounters issues like torque ripples and reduced power quality due to a hysteresis controller. In response, the study proposes an innovative DPC method for DFIGs using artificial neural networks (ANNs). Experimental verification shows ANNs effectively addressing issues with the hysteresis controller and switching table. Additionally, the study addresses wind speed variability by employing an artificial neural network to directly control reactive and active power of DFIG, aiming to minimize challenges with varying wind speeds. Results highlight the effectiveness and reliability of the developed intelligent strategy, outperforming traditional methods by reducing current harmonics and improving dynamic response. This research contributes valuable insights into enhancing the performance and reliability of renewable energy systems, advancing solutions for wind energy integration complexities.

## 1. Introduction

Today, the significance of generating electricity from renewable energy systems is steadily increasing due to the escalating shortage of conventional energy sources and the pressing issue of global warming. Furthermore, the utilization of carbon accounting serves as a crucial technique for estimating the amount of carbon emissions produced by an organization, particularly in relation to electricity delivery. This is especially relevant as more industries and individuals actively participate in carbon reduction initiatives [1]. Additionally, the ability to limit the rise in global temperature to 1.5°C and achieve the CO2 reduction targets by 2050 may rely on the widespread adoption of renewable energy sources (RESs) coupled with increased electrification [2]. In light of advancements in technology, wind energy has emerged as one of the most promising renewable energy sources worldwide, primarily due to its remarkable efficiency and adaptable control capabilities [3].

The conversion of wind energy into mechanical energy within the Wind Energy Conversion System (WECS) necessitates subsequent conversion into electrical energy. This conversion process mandates a harmonious interaction between the mechanical turbine and the electrical generator, emphasizing the need for both an electrical control system for the generator and a mechanical control system for the turbine to ensure reliability and efficiency [4]. However, a critical consideration prior to the development of the controller is the appropriate selection of the generator. Constant-speed operation, despite its drawbacks such as low conversion efficiency, direct impact of varying wind speeds on electrical utility, and high sensitivity to voltage drops and grid faults [5], has been surpassed by the adoption of variable-speed wind generators [6]. Various types of generators are used in wind energy production, including asynchronous, synchronous, DC, and doubly-fed induction generator (DFIG) [7–10]. Presently, the most prevalent generator type employed in wind turbine systems is the doubly-fed induction generator [11–13], which offers an extensive range of advantages [14]. In this system, the rotor windings of the generator are connected to the power grid via an AC-DC-AC converter, while the stator windings are interconnected in series with the grid.

Integration wind energy systems into the power grid can create difficulties due to the unpredictable nature of wind speed. These fluctuations lead to issues such as power oscillations and inconsistencies in energy generation, which can have adverse ripple effects on the electromagnetic torque of the generator. Based on existing literature, researchers express significant concern about enhancing this conversion process. For instance, some focus on increasing the level of wind penetration [15, 16], while others explore the option of utilizing a battery charge controller for energy storage [17–19]. On the other hand, several control approaches have been proposed to enhance the performance of Wind Turbine Systems (WTS) based on Doubly-Fed Induction Generators (DFIG) during normal operations [20]. Among these approaches, two prominent control techniques for DFIG are field-orientation control (FOC) and direct power control (DPC) [21]. Field-orientation control, implemented using a proportional integral (PI) controller, aims to decouple the variables of the machine, making it resemble a direct-current generator [22]. This technique continues to be widely utilized due to its clarity and straightforward implementation [23]. Furthermore, the design of PI parameters significantly influences the dynamic performance of DFIG under FOC. In many applications of Wind Energy Conversion Systems (WECS), the classical PI controller demonstrates satisfactory performance. For instance, in [4], a closed-loop PI controller is employed to adjust the mechanical transfer function’s performance parameters, ensuring stability and accuracy. However, the practical effectiveness of this control strategy is limited due to reduced robustness and an inability to adapt to changes in machine parameters caused by factors like grid voltage drops, model inaccuracies, and unexpected variables such as temperature variations. To address these challenges and enhance the robustness of WECS, various advanced control strategies have been proposed as alternatives to FOC, including sliding mode control (SMC) [24] and backstepping control (BSC) [25]. The paper in [24] employs sliding mode control to enhance the performance of variable-speed wind turbine driving a DFIG. While sliding mode control (SMC) has some advantages such as robustness, simplicity, and fast response, it also comes with disadvantages, including the chattering effect and parameter sensitivity, which can limit the performance and applicability for DFIG control [26, 27]. Consequently, modification and improvements of the SMC technique have been proposed in the literature, such as the super-twisting algorithm [28]. In a different approach the paper in [25] proposes and tests the nonlinear Backstepping control for wind turbine systems. This technique offers benefits such as improved performance, ease of installation, and resilience to external disturbances. However, its drawbacks include complexity and sensitivity. To address these issues, the literature suggests specific improvements and alterations to the backstepping technique, such as adaptative backstepping control [29]. Direct power control (DPC), introduced in 1998 by Neghouchi [30], is another linear control methods widely adopted in various applications, emerging as a competitor to vector control. DPC provides a solution to the challenges posed by sensitivity to parametric variation. Research reveals that DPC significantly enhances dynamic response, allowing for rapid and precise control of generated power [31]. Furthermore, DPC proves effective in minimizing torque and power oscillations within the generator, resulting in a smoother and more stable operational performance [32]. The study demonstrates that the implementation of DPC contributes to improved energy conversion efficiency, optimizing the overall performance of DFIG systems [33]. Additionally, the analysis highlights the simplicity of the control structure employed by dpc, presenting a contrast to traditional vector control methods. This simplicity not only facilitates easier implementation but also contributes to the maintenance of the system [34]. Moreover, the study underscores DPC’ notable feature of reduced sensitivity to variations in system parameters, enhancing the robustness and reliability of the DFIG system [31]. However, Direct Power Control (DPC) technique has two main drawbacks: significant power fluctuations and varying commutation frequencies. Various solutions have been proposed to enhance the efficiency and characteristics of Direct Power Control (DPC). One approach involves integrating DPC with various other controllers, including nonlinear controllers and artificial intelligence algorithms. In references [20, 35], authors integrated DPC with sliding mode control (SMC) to overcome the drawbacks of DPC. Reference [20] eliminates the use of hysteresis controllers and switching tables, leading to increased complexity in the implementation process. As a result, adopting the DPC strategy becomes more costly and requires more experimentation. Another proposed solution includes the use of a backstepping controller to enhance the quality of the output current in DFIG system [20–36]. However, the inclusion of a backstepping controller amplifies the complexity and execution challenges associated with Direct Power Control. Additionally, reference [37] presented DPC for a matrix converter feeding a DFIG with a fixed switching frequency. Another novel DPC approach for DFIG, discussed in [38], utilizes L-filter to enhance the quality of DFIG-WTS currents and reduce current, torque, and active power fluctuations. In [39], a combination of fuzzy logic and genetic algorithm is employed to minimize power ripples and enhance the dynamic response of DPC.

In this paper, we address overcome the drawbacks of DPC by employing a combination of Artificial Neural Network (ANN) and the Pulse Width Modulation (PWM) technique for real wind profiles. Artificial Neural Networks (ANNs) serve as intelligent controllers in nonlinear systems, offering several advantages across applications. These include handling large datasets, exhibiting flexibility and nonlinearity, enabling automatic procedures and multitasking, and facilitating rapid processing [40]. The work presented by the author in [41] introduces a novel approach that utilizes an artificial neural network to improve the convergence trajectory and minimize tracking errors in super-twisting sliding mode control. Additionally, in [42], neural networks are applied for stable control of a nonlinear DFIG in wind power systems. The benefits of ANN-based controllers are evident, including reduced peak amplitudes during transient regimes and faster system responses, resulting a shorter time to reach a steady state. Therefore, ANN proves to be a well-established data processing technique, enhancing the understanding and mastery of the control strategy. The intelligent Direct Power Control (DPC) strategy, incorporating Pulse Width Modulation (PWM), is employed to regulate the Rotor-Side Converter (RSC). This customized DPC strategy significantly differs from the traditional approach by eliminating the conventional switching table and comparators, resulting in a more efficient DPC strategy.

This study entails an experimental investigation of an intelligent Direct Power Control (DPC) strategy, employing an Artificial Neural Network (ANN) controller to enhance current quality and reduce ripples in the Doubly Fed Induction Generator Wind Turbine System (DFIG-WTS). This innovative DPC approach deviates significantly from the conventional method by replacing both the switching table and hysteresis controller, resulting in a more robust DPC strategy.

The main contributions of this paper can be summarized as follows:

- Introduction of an innovative DPC strategy that incorporates artificial neural networks and pulse width modulation (PWM) technique.
- Overcoming the drawbacks and issues associated with DPC.
- Utilization of real-time wind speed data to ensure a reliable basis for analysis.
- Mitigation of ripples in active power and current, leading to an overall enhancement in the performance of the DFIG-WTS.

The paper is structured as follows:

In Section 2, we present a wind energy conversion system model, which is divided into two subsections: wind turbine modeling and double-fed induction generator modeling.

Section 3, formulates the problem by explaining the control configuration of DPC-Classic and outlining the control objectives to be achieved with the intelligent DPC controller.

In Section 4 we describe the implementation of artificial neural network control (ANN) for operating the system, enabling control of active and reactive power.

Section 5 utilizes a real-world profile to analyze experimental results, conducting a comparative study to assess the performance of the two controllers proposed in this work, along with other publications.

Finally, in section 6 we provide a conclusion.

## 2. Wind energy conversion system modelling

### 2.1. Wind turbine (HAWT) modelling

The wind turbine plays a crucial role in the wind energy conversion system (WECS) as it transforms kinetic energy into mechanical energy. This component is connected to the generator via a gearbox, which amplifies the rotational speed from the wind turbine to the electric generator [43–51]. To transmit the generator’s output to the electrical grid, a control system is utilized [52, 53]. Eq (1) represents the mechanical power *P*_*m*_ generated by the wind turbine [54]:

$$P_{m}=\frac{1}{2}C_{p}(\lambda ,\beta )\rho \pi R^{2}V_{wind}^{3}$$ (1)

Where *ρ* is the air density, *R* is the rotor radius, *V*_*wind*_ is the wind speed, and *C*_*p*_*(λ*, *β)* is the power coefficient, which depends on two aspects: *β* the blade pitch angle and λ the tip speed ratio, which is defined as:

$$\lambda =\frac{\Omega_{turbine}R}{V_{wind}}$$ (2)

Ω_*turbine*_ is the angular speed of turbine, related to the mechanical speed of generator Ω_*mec*_ by the following equation:

$$\Omega_{mec}=G_{r}\Omega_{turbine}$$ (3)

With *G*_*r*_ is the gearbox ratio

Eq (4) depicts the equation of motion that relates the torque and the speed

$$J_{total}\frac{d\Omega_{mec}}{dt}=T_{mec}=T_{g}-T_{em}-f\Omega_{mec}$$ (4)

Where *J*_*total*_ is the total mechanical inertia, *f* is the coefficient of the friction, and *T*_*mec*_ *T*_*g*_, *T*_*em*_ are successively the mechanical torque, the torque of DFIG, the electromagnetic torque.

In this paper, the model of the power coefficient is defined as [53, 54]:

$$[\begin{array}{l} C_{p}(\lambda ,\beta )=C_{1}(\frac{C_{2}}{\lambda_{i}}-C_{3}.\beta –C_{4})\cdot e^{\left(\frac{-C_{5}}{\lambda_{i}}\right)}+C_{6}\lambda \\ \frac{1}{\lambda_{i}}=\frac{1}{\lambda +0.08\beta }-\frac{0.035}{\beta^{3}+1} \end{array}$$ (5)

With $C_{1}=0.5176,\;C_{2}=116,\;C_{3}=0.4,\;C_{4}=5,\;C_{5}=21,\;C_{6}=0.0068$ To enable the generator to operate at its optimal speed, it is necessary to ensure that the wind speed is optimal. This can be achieved by utilizing the maximum power point (MPPT), where the power coefficient is kept at its maximum [55].

**Fig 1:**
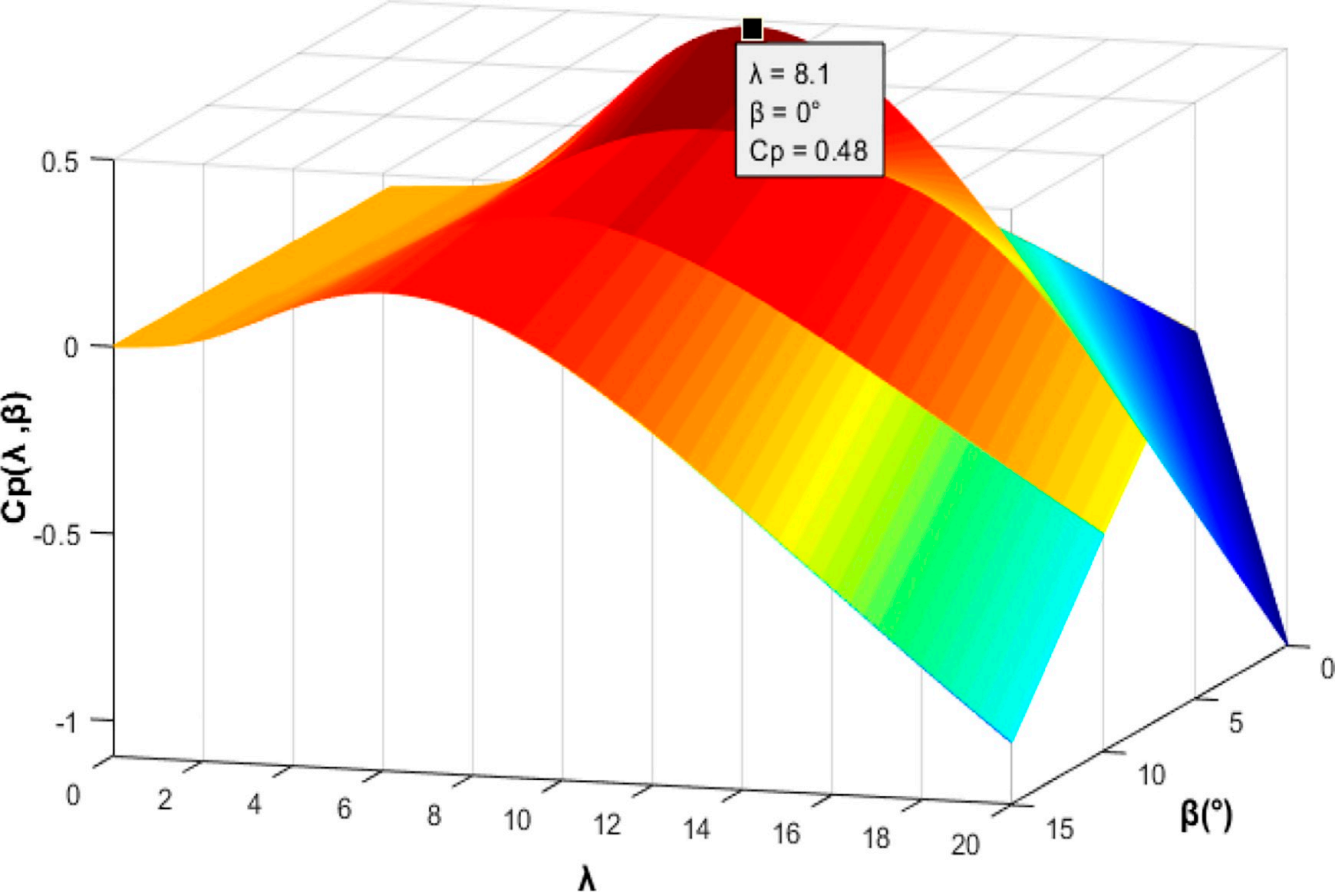
2D plot of Cp(λ, β).

Fig 1 shows the calculated values of *C*_*p*_ with respect to *λ* and *β*. The maximum value of *C*_*p*_ is obtained by fixing *λ* and *β* to their optimal values. It is observed in Fig 1 below that $C_{p}{\left(\lambda ,\beta \right)}_{\max}=0.48$ for *λ* = 8.1 and *β* = 0°.

### 2.2. Doubly fed induction generator (DFIG) modelling

**Fig 2:**
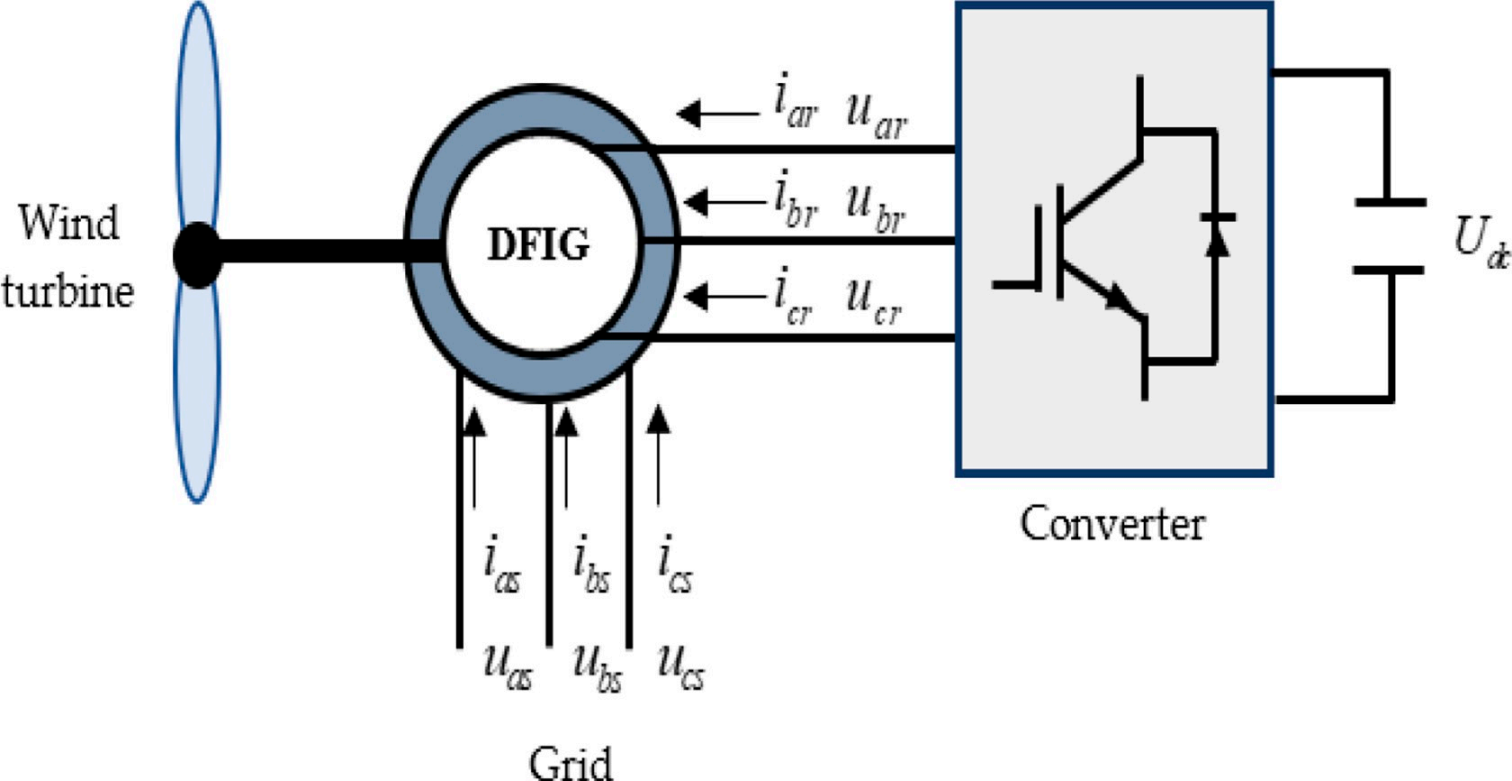
Structure of the DFIG for (WPG).

The Doubly Fed Induction Generator (DFIG) features two sets of three-phase windings with mutual *L*_*m*_ and self-inductances *L*_*ss*_, *L*_*rr*_ [56]. The structure of the DFIG for wind power generation (WPG) is illustrated in Fig 2, where *u*_*s*_ and *u*_*r*_ are the stator and rotor voltages, *i*_*s*_ and *i*_*r*_ are the stator and rotor currents, and *U*_*dc*_ is the direct bus voltage.

The voltage equations for the three-phase stator are [52]:

$$E_{1}\{\begin{array}{l} u_{as}=R_{s}i_{as}+\frac{d\Phi_{as}}{dt}\\ u_{bs}=R_{s}i_{bs}+\frac{d\Phi_{bs}}{dt}\\ u_{cs}=R_{s}i_{cs}+\frac{d\Phi_{cs}}{dt} \end{array}\;\mathrm{Where}\;E_{2}\{\begin{array}{l} \Phi_{as}=L_{ss}i_{as}+L_{m}i_{ar}\\ \Phi_{bs}=L_{ss}i_{bs}+L_{m}i_{br}\\ \Phi_{cs}=L_{ss}i_{cs}+L_{m}i_{cr} \end{array}$$ (6)

Similarly for the rotor [45]:

$$E_{3}\{\begin{array}{l} u_{ar}=R_{r}i_{ar}+\frac{d\Phi_{ar}}{dt}\\ u_{br}=R_{r}i_{br}+\frac{d\Phi_{br}}{dt}\\ u_{cr}=R_{r}i_{cr}+\frac{d\Phi_{cr}}{dt} \end{array}\;\mathrm{Where}\;E_{4}\{\begin{array}{l} \Phi_{ar}=L_{rr}i_{ar}+L_{m}i_{as}\\ \Phi_{br}=L_{rr}i_{br}+L_{m}i_{bs}\\ \Phi_{cr}=L_{rr}i_{cr}+L_{m}i_{cs} \end{array}$$ (7)

During the rotation of the machine, the mutual inductances and the angle between the rotor and stator circuits vary, leading to a dynamic mathematical model [57]. To simplify the mathematical representation, a two-axis model (dq) can be employed, which is less complex than the three-axis model (abc). By utilizing Park’s model, the system can be transformed from a three-phase representation to a direct and quadrature representation.

Utilizing the Park transformation (*T*_*s*_), the stator voltage equations can be converted to a dq coordinate system in a synchronous reference frame, resulting in the following equations:

$$T_{s}(E_{1})\{\left[\begin{array}{l} v_{ds}\\ v_{qs} \end{array}]=T_{s}[\begin{array}{l} v_{as}\\ v_{bs}\\ v_{cs} \end{array}]=R_{s}T_{s}[\begin{array}{l} i_{as}\\ i_{bs}\\ i_{cs} \end{array}]+T_{s}\frac{d}{dt}[\begin{array}{l} \Phi_{as}\\ \Phi_{bs}\\ \Phi_{cs} \end{array}\right]$$ (8)

$$E_{5}\{\begin{array}{l} v_{ds}=R_{s}i_{ds}+\frac{d}{dt}\Phi_{ds}-\omega_{s}\Phi_{qs}\\ v_{qs}=R_{s}i_{qs}+\frac{d}{dt}\Phi_{qs}+\omega_{s}\Phi_{ds} \end{array}\;\mathrm{Where}\;E_{6}\{\begin{array}{l} \Phi_{ds}=L_{ss}i_{ds}+L_{m}i_{dr}\\ \Phi_{qs}=L_{ss}i_{qs}+L_{m}i_{qr} \end{array}$$ (9)

Similarly, the rotor voltage equations given in *E*_3_ can be transformed into the dq frame by Park transformation (*T*_*r*_), resulting in the new equations of the rotor voltage:

$$T_{r}(E_{3})\{\left[\begin{array}{l} v_{dr}\\ v_{qr} \end{array}]=T_{r}[\begin{array}{l} v_{ar}\\ v_{br}\\ v_{cr} \end{array}]=R_{r}T_{r}[\begin{array}{l} i_{ar}\\ i_{br}\\ i_{cr} \end{array}]+T_{r}\frac{d}{dt}[\begin{array}{l} \Phi_{ar}\\ \Phi_{br}\\ \Phi_{cr} \end{array}\right]$$ (10)

$$E_{7}\{\begin{array}{l} v_{dr}=R_{r}i_{dr}+\frac{d}{dt}\Phi_{dr}-\omega_{r}\Phi_{qr}\\ v_{qr}=R_{r}i_{qr}+\frac{d}{dt}\Phi_{qr}+\omega_{r}\Phi_{dr} \end{array}\;\mathrm{Where}\;E_{8}\{\begin{array}{l} \Phi_{dr}=L_{rr}i_{dr}+L_{m}i_{ds}\\ \Phi_{qr}=L_{rr}i_{qr}+L_{m}i_{qs} \end{array}$$ (11)

*ω*_*s*_ and *ω*_*r*_ are the stator and the rotor currents pulsations.

The real and the reactive powers of the DFIG are [58]:

$$E_{9}\{\begin{array}{l} P_{s}=\frac{3}{2}(v_{ds}i_{ds}+v_{qs}i_{qs})\\ Q_{s}=\frac{3}{2}(v_{qs}i_{ds}-v_{ds}i_{qs}) \end{array}$$ (12)

Eq 13 establishes a relationship between the mechanical and electrical aspects of the machine through the electromagnetic torque [59].


$$T_{em}=\frac{3}{2}p\frac{L_{m}}{L_{ss}}(\Phi_{qs}i_{dr}-\Phi_{ds}i_{qr})$$ (13)

The vector control by oriented stator field (Φ_*ds*_ = Φ_*s*_ and Φ_*qs*_ =0) is considered a way to simplify the modeling of the machine because it assures dissociation between its variables and creates a model that resembles a DC machine. This method allows for the decoupling of the stator’s active and reactive powers. Supposing that Φ_*sd*_ is constant at the permanent regime and *R*_*s*_ is neglected [60], consequently:

$$E_{10}\{\begin{array}{l} v_{ds}=0\\ v_{qs}=v_{s}=\omega_{s}\Phi_{s} \end{array}$$ (14)

$$E_{11}\{\begin{array}{l} \Phi_{s}=L_{ss}i_{ds}+L_{m}i_{dr}\\ 0=L_{ss}i_{qs}+L_{m}i_{qr} \end{array}\;➔\;E_{12}\{\begin{array}{l} i_{ds}=\frac{\Phi_{s}}{L_{ss}}-\frac{L_{m}}{L_{rr}}i_{dr}\\ i_{qs}=-\frac{L_{m}}{L_{ss}}i_{qr} \end{array}$$ (15)

Replacing *E*_12_ in *E*_8_:

$$E_{13}\{\begin{array}{l} \Phi_{dr}=\sigma L_{rr}i_{dr}+\frac{L_{m}}{L_{ss}}\Phi_{s}\\ \Phi_{qr}=\sigma L_{rr}i_{qr} \end{array}\;➔\;E_{14}\{\begin{array}{l} i_{dr}=\frac{\Phi_{dr}}{\sigma L_{rr}}-\frac{L_{m}}{\sigma L_{rr}L_{ss}}\Phi_{s}\\ i_{qr}=\frac{\Phi_{qr}}{\sigma L_{rr}} \end{array}\;With\;\sigma =1-\frac{L_{m}^{2}}{L_{ss}L_{rr}}$$ (16)

Replacing *E*_12_ in *E*_9_ and *E*_14_ in *E*_15_:

$$E_{15}\{\begin{array}{l} P_{s}=-\frac{3}{2}\frac{L_{m}}{L_{ss}}v_{s}i_{qr}\\ Q_{s}=\frac{3}{2}v_{s}(\frac{\Phi_{s}}{L_{ss}}-\frac{L_{m}}{L_{ss}}i_{dr}) \end{array}\;➔\;E_{16}\{\begin{array}{l} P_{s}=-\frac{3}{2}\frac{L_{m}}{\sigma L_{rr}L_{ss}}v_{s}\Phi_{qr}\\ Q_{s}=\frac{3}{2}v_{s}(\frac{\Phi_{s}}{\sigma L_{ss}}-\frac{L_{m}}{\sigma L_{rr}L_{ss}}\Phi_{dr}) \end{array}$$ (17)

The electromagnetic torque and the rotor voltages are:

$$T_{em}=-\frac{3}{2}p\frac{L_{m}}{L_{ss}}\Phi_{s}i_{qr}$$ (18)

$$E_{17}\{\begin{array}{l} v_{dr}=R_{r}i_{dr}+\sigma L_{r}i_{dr}^{\cdot }+e_{qr}\\ v_{qr}=R_{r}i_{qr}+\sigma L_{r}i_{qr}^{\cdot }+e_{dr}+e_{\Phi } \end{array}\;with\{\begin{array}{l} e_{qr}=-\omega_{r}\sigma L_{r}i_{qr}\\ e_{dr}=\omega_{r}\sigma L_{r}i_{dr}\\ e_{\Phi }=\omega_{r}\frac{L_{m}}{L_{s}}\Phi_{s} \end{array}$$ (19)

## 3. Problem formulation

### 3.1 Control configuration

**Fig 3:**
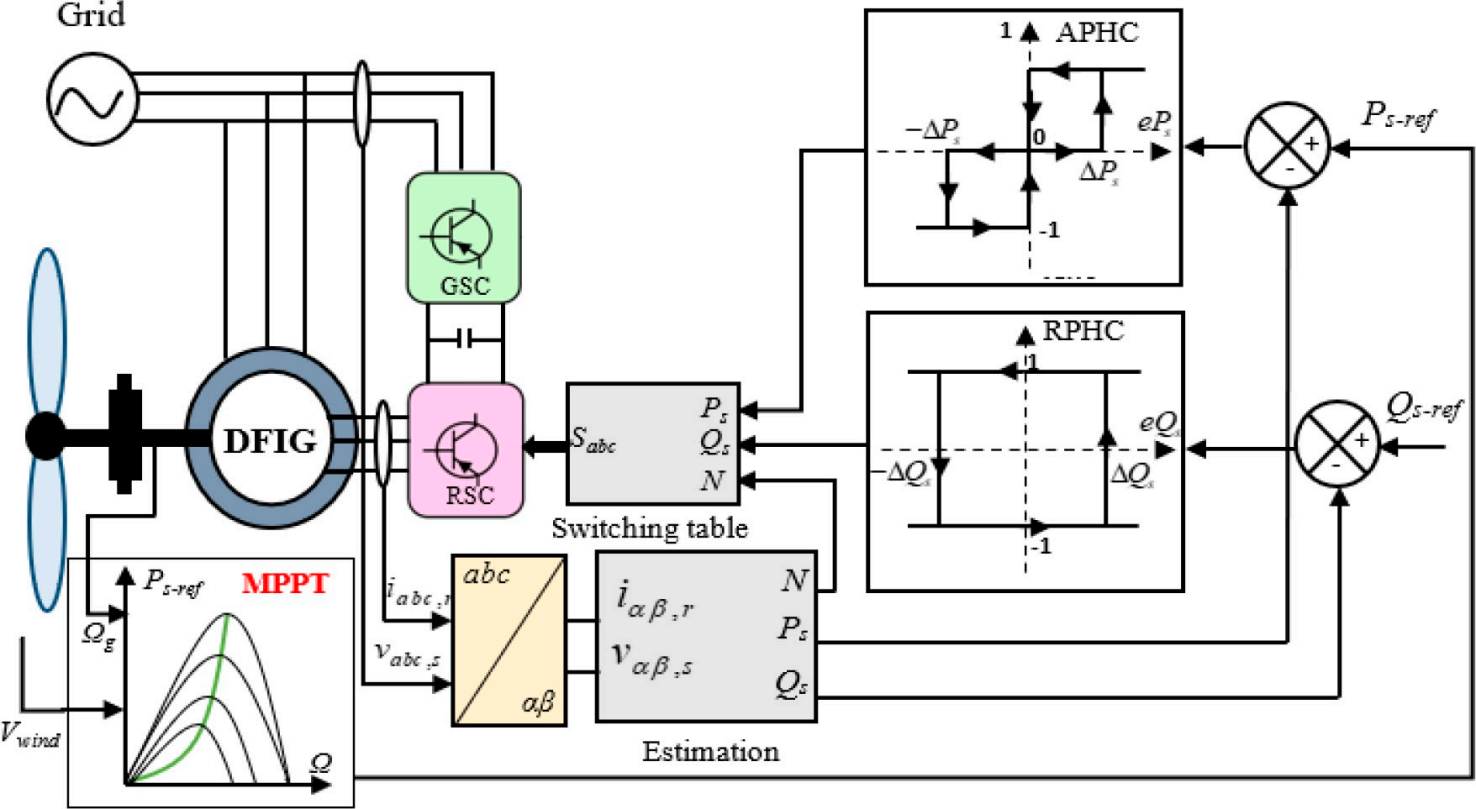
DPC-Classic strategy.

In this paper, the assessment of power performance employs the DPC-Classic strategy instead of repetitive control configurations. DPC offers several advantages, including rapid dynamic response, reduced dependence on machine models, simplified implementation, and low computational complexity [57, 61]. Fig 3 illustrates the conventional DPC approach for controlling wind turbine systems driven by a Doubly Fed Induction Generator (DFIG). Two hysteresis comparators (APHC, RPHC) directly control the DFIG machine’s stator active and reactive powers, while a switching table is utilized for the converter on the rotor side (RSC).

As observed in the preceding figures, the DPC technique necessitates selecting an optimal voltage vector from a switching table to minimize and regulate the discrepancies between the measured and reference powers within predefined hysteresis bands [62].

The hysteresis controller changes its output to “1” if the error of the power (eP_s_ or eQ_s_) reaches a more elevated level [63], also changes its output to “-1” if *eP*_*s*_≤−Δ*P*_*s*_ or *eQ*_*s*_≤−Δ*Q*_*s*_, and to “0” if −Δ*P*_*s*_≤*eP*_*s*_≤Δ*P*_*s*_

Equation E_16_ from the previous section demonstrates that we can determine the relationship of the P_S_ and Q_s_ as functions of two rotor flux components in the reference frame (α_r_-β_r_) that revolve with the DFIG’s rotor. Therefore:

$$\left\{ \begin{array}{l} P_{s}=-\frac{3}{2}\frac{L_{m}}{\sigma L_{rr}L_{ss}}v_{s}\Phi_{\beta r}\\ Q_{s}=\frac{3}{2}v_{s}(\frac{\Phi_{s}}{\sigma L_{ss}}-\frac{L_{m}}{\sigma L_{rr}L_{ss}}\Phi_{\alpha r}) \end{array}\;\mathrm{Where}\;\{\begin{array}{l} \Phi_{\alpha r}=\sigma L_{rr}i_{\alpha r}+\frac{L_{m}}{L_{ss}}\Phi_{s}\\ \Phi_{\beta r}=\sigma L_{rr}i_{\beta r}\\ |\Phi_{s}|=\frac{\left|\bar{v_{s}}\right|}{\omega_{s}} \end{array}\right.$$ (20)

The following expressions show that the active and reactive powers depend directly on the relative angle *δ* between the stator and rotor flux vectors and their amplitude. Therefore, by modifying *δ*, it is possible to control P_s_ and Q_s_ [57].


$$\left\{ \begin{array}{l} P_{s}=-\frac{3}{2}\frac{L_{m}}{\sigma L_{rr}L_{ss}}\omega_{s}|\Phi_{s}||\Phi_{r}|\sin\delta \\ Q_{s}=\frac{3}{2}\frac{\omega_{s}|\Phi_{s}|}{\sigma L_{ss}}(\frac{L_{m}}{L_{rr}}|\Phi_{r}|\cos\delta -|\Phi_{s}|) \end{array}\;➔\;\{\begin{array}{l} \frac{dP_{s}}{dt}=-\frac{3}{2}\frac{L_{m}\omega_{s}}{\sigma L_{rr}L_{ss}}|\Phi_{s}|\frac{d(|\Phi_{r}|\sin\delta )}{dt}\\ \frac{dQ_{s}}{dt}=\frac{3}{2}\frac{L_{m}\omega_{s}}{\sigma L_{rr}L_{ss}}|\Phi_{s}|\frac{d(|\Phi_{r}|\cos\delta )}{dt} \end{array}\right.$$ (21)

To select the optimum rotor voltage vector, the relative position of the rotor flux must be determined. Due to the concern with a more stringent control, the evolution space of Φ_*r*_ in the reference frame under consideration is divided into sex sectors for this purpose [63]. A selection of appropriate vectors applied to the converter on the RSC is shown in Table 1 below. This table enables the rotor flux and powers to be controlled [64, 65].

**Table 1:** Selection table of optimal vectors (active and reactive power).

		RPHC	1			-1		
		APHC	1	0	-1	1	0	-1
Sector N concerning the angle δ	1	(330°, 30°)	V_5_	V_7_	V_3_	V_6_	V_0_	V_2_
	2	(30°, 90°)	V_6_	V_0_	V_4_	V_1_	V_7_	V_3_
	3	(90°, 150°)	V_1_	V_7_	V_5_	V_2_	V_0_	V_4_
	4	(150°,210°)	V_2_	V_0_	V_6_	V_3_	V_7_	V_5_
	5	(210°,270°)	V_3_	V_7_	V_1_	V_4_	V_0_	V_6_
	6	(270°,330°)	V_4_	V_0_	V_2_	V_5_	V_7_	V_1_

V_0_ = [0,0,0]; V_1_ = [1,0,0]; V_2_ = [1,1,0]; V_3_ = [0,1,0]; V_4_ = [0,1,1]; V_5_ = [0,0,1]; V_6_ = [1,0,1]; V_7_ = [1,1,1]

### 3.2 Control objectives

The direct power control (DPC) strategy has been widely utilized and extensively discussed in DFIG control applications due to its numerous advantages [66, 67]. It effectively decouples active and reactive power, exhibits prompt response without overshoot, and maintains minimal static error, among other benefits. However, the traditional DPC approach suffers from drawbacks such as a high switching frequency, resulting in significant harmonic distortion in the generated currents and poor current quality, leading to various faults [20, 28–45]. This not only increases maintenance costs but also poses a risk to the grid. Additionally, there are considerable ripples in active and reactive powers due to the variable switching frequency employed in this strategy [68, 69].

To overcome these limitations, this work proposes a novel control method based on Artificial Neural Networks (ANN) [70, 71]. The suggested control approach aims to retain the advantages of the conventional DPC while simultaneously minimizing the THD of current and reducing the ripples in active and reactive powers.

## 4. System control

Research in neural computing has extensively explored the application of Artificial Neural Networks (ANN) as a data processing technique [53]. This method extends the capabilities of traditional nonlinear automation techniques, providing enhanced effectiveness and reliability in various applications, including identification, control, and filtering. Artificial neurons within ANNs progressively improve their accuracy by learning from input data. Once adequately trained, they can generate results that closely match the desired outcomes [72].

**Fig 4:**
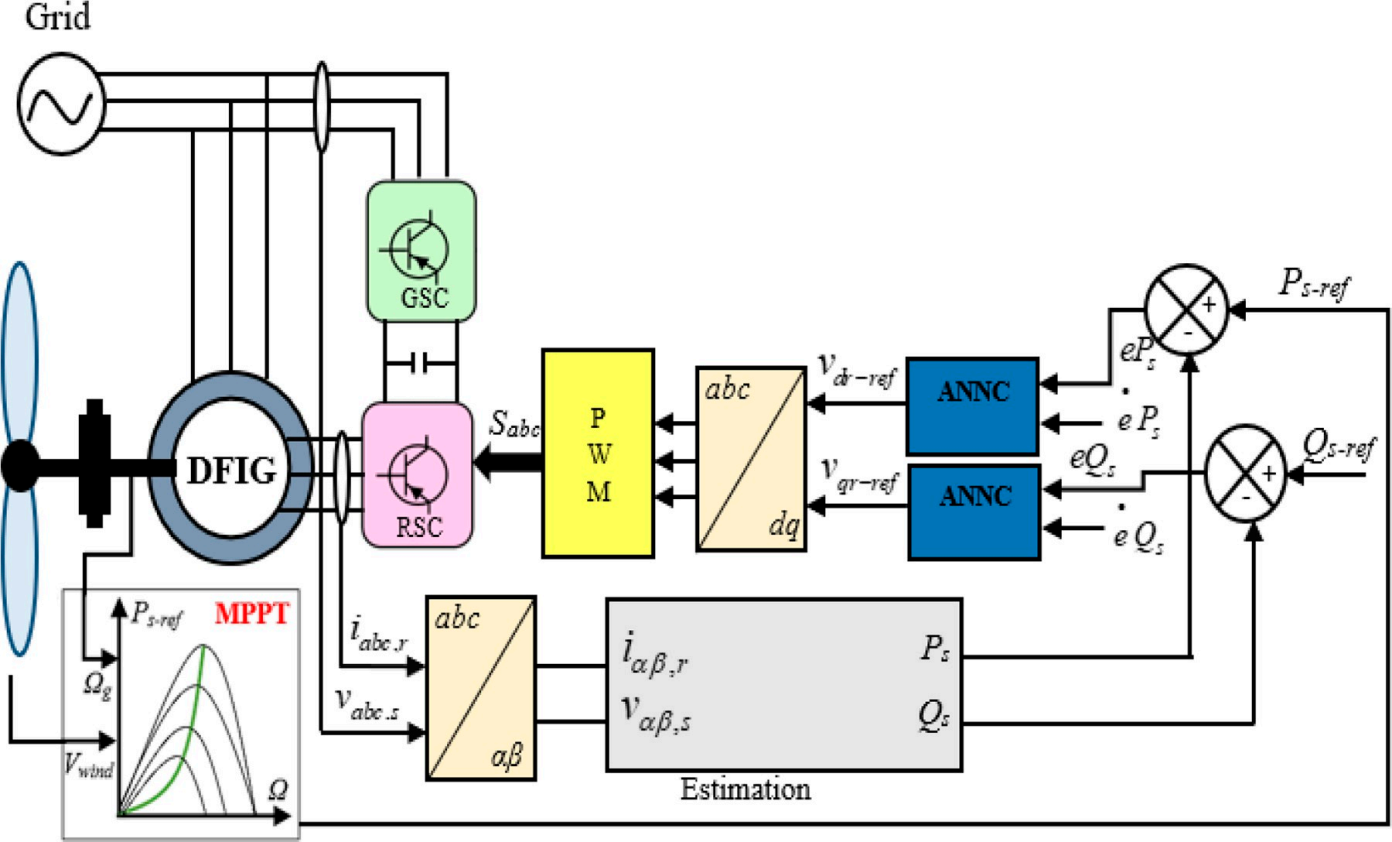
DPC-ANN strategy.

**Fig 5:**
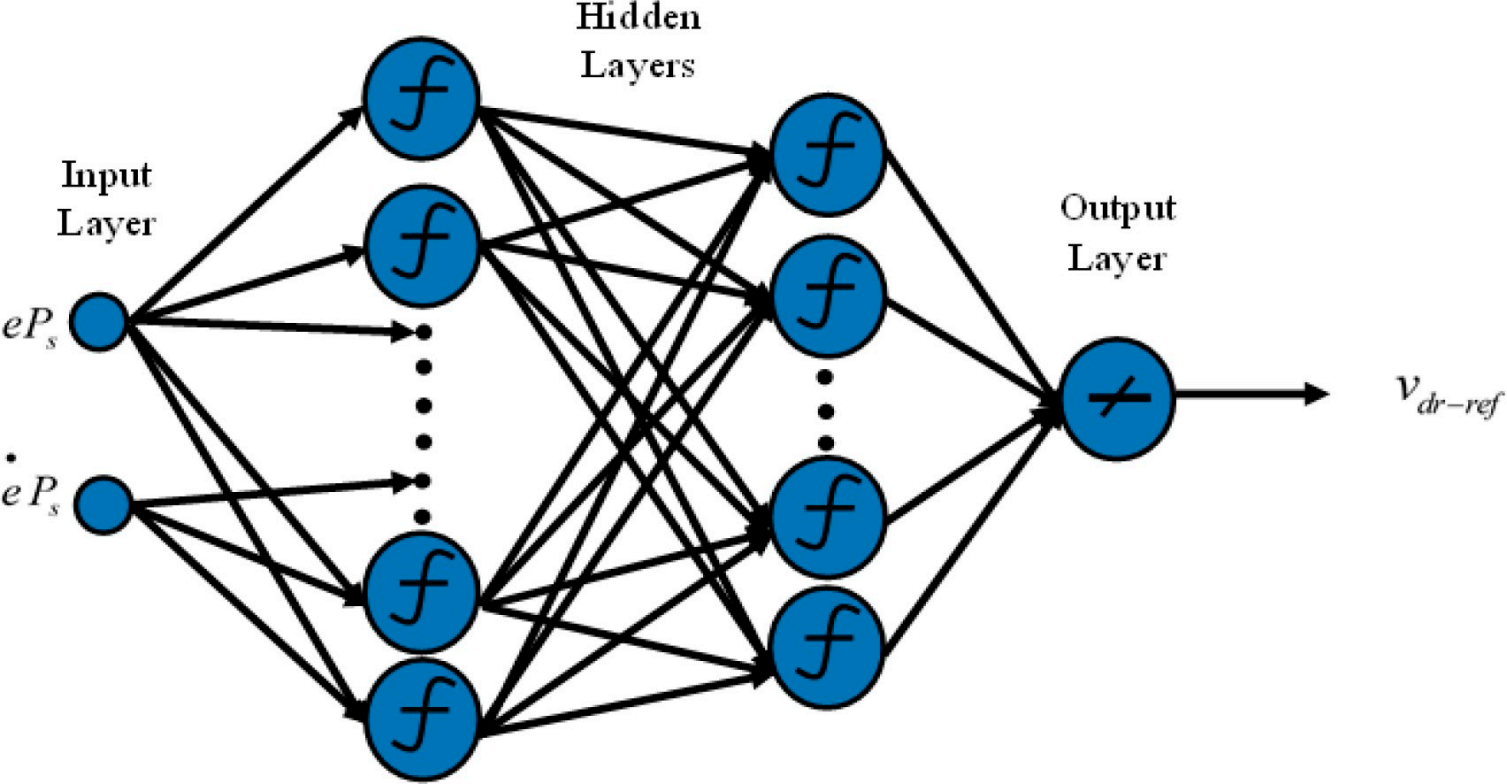
Neural network training (a) and mean squared error (b) for the active power controller.

**Fig 6:**
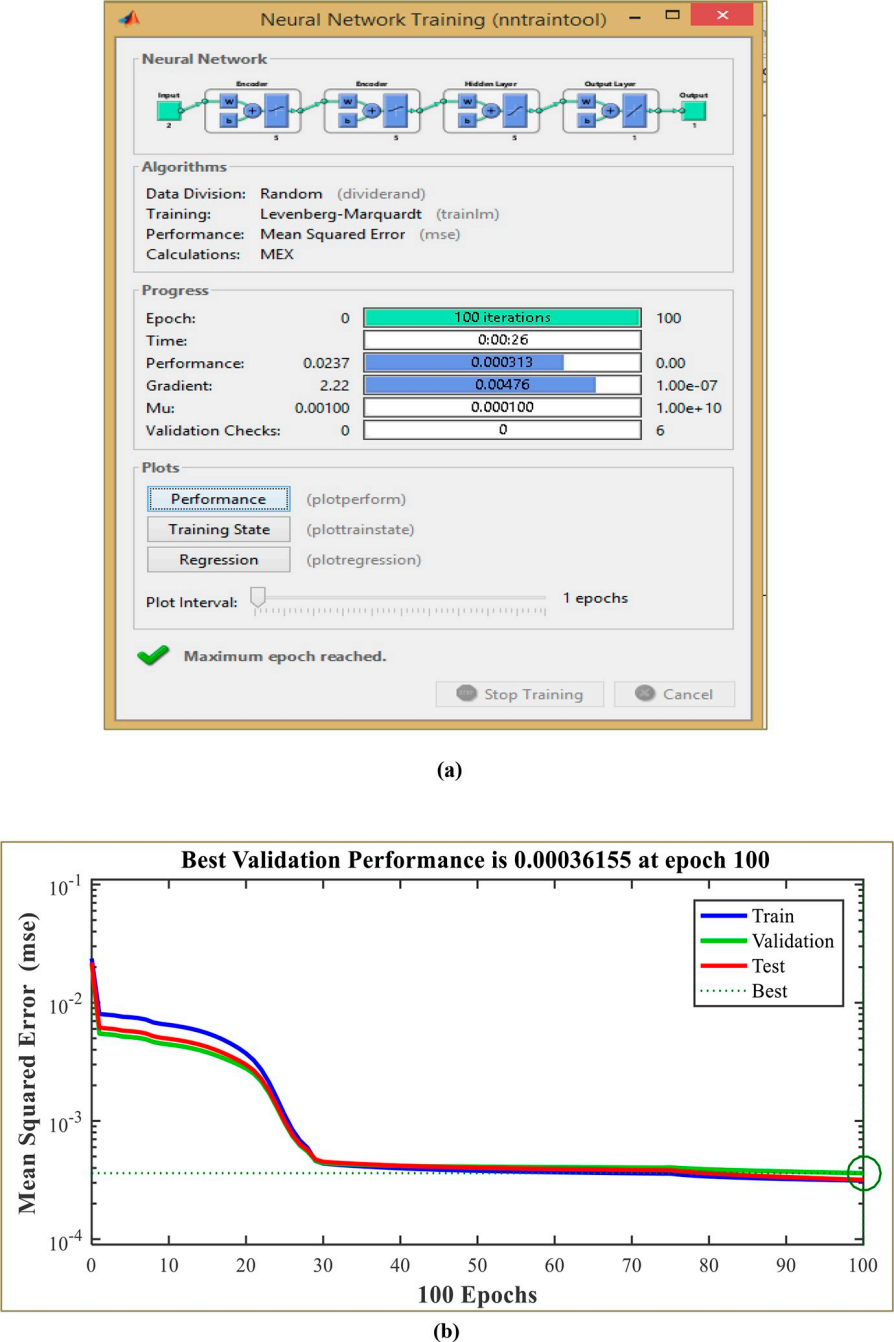
Neural network training (a) and mean squared error (b) for the reactive power.

In this research, the classical DPC’s hysteresis comparators and switching table are replaced with two neural multilayer perceptron controllers (MLP) and the pulse width modulation technique (PWM). The Levenberg-Marquardt (LM) backpropagation algorithm is employed for the learning process. Fig 4 illustrates the implementation of intelligent DPC using an artificial neural network controller (ANNC) for controlling wind turbine systems with a DFIG. The parameters of the ANN controller presented in this paper can be found in the S1 Appendix. Figs 5 and 6 showcase the learning progress of the active and reactive power controllers. The proposed structure (2-5-5-5-1) for the controllers of active and reactive powers achieves optimal performance quickly, reaching the best results by the 30th and 50th iterations, respectively. Moreover, in Figs 5B and 6B, the Mean Square Error (MSE) progression is depicted concerning the number of iterations for the reactive and active power controllers, respectively. Notably, both controllers achieve a low MSE BY the 100^th^ iteration.

### 4.1 Artificial neural networks controller for active power

**Fig 7:**
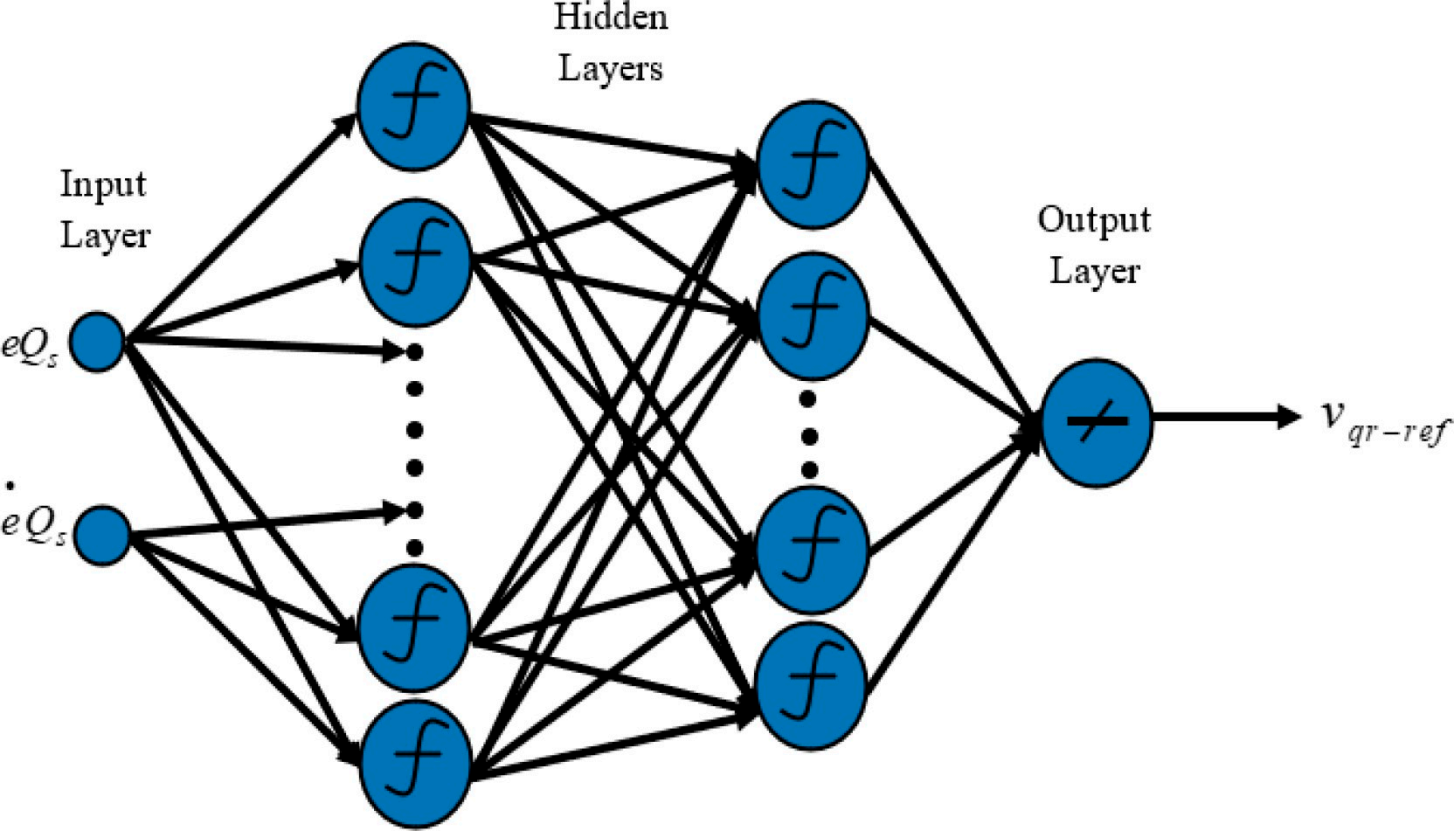
The architecture of the proposed MLP-ANN controller for active power.

The neural network takes the active power error and its derivatives as inputs, producing the rotor voltage reference as an output. To identify the optimal architecture of the network, the initial MLP controller utilized two hidden layers, as depicted in Fig 7.

### 4.2 Artificial neural networks controller for reactive power

**Fig 8:**
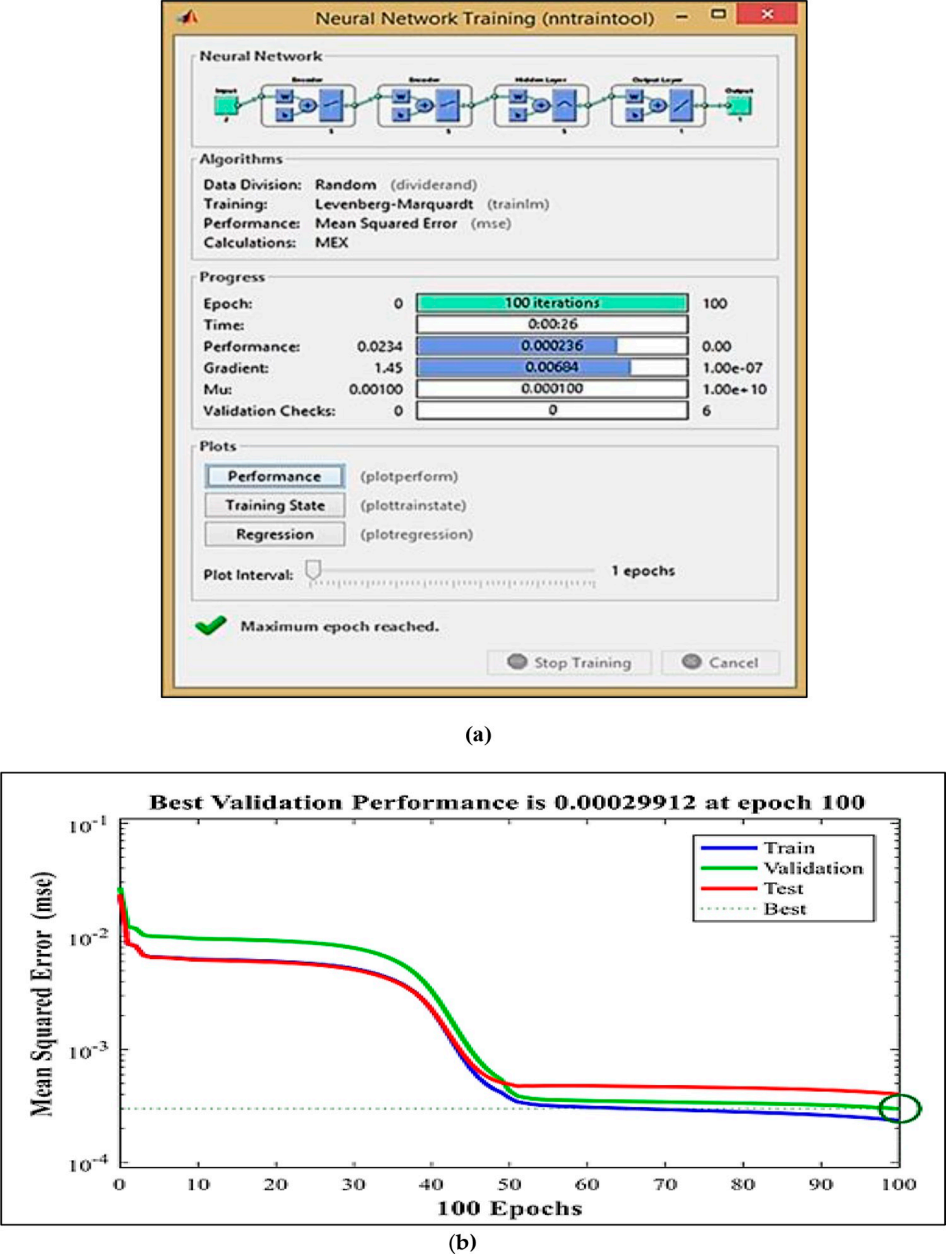
The architecture of the proposed MLP-ANN controller for reactive power.

The neural network takes the reactive power error and its derivatives as inputs, generating the rotor voltage reference as the output. To identify the optimal architecture of the network, the second MLP controller was designed with two hidden layers, as illustrated in Fig 8.

## 5. Simulation results and discussions

**Fig 9:**
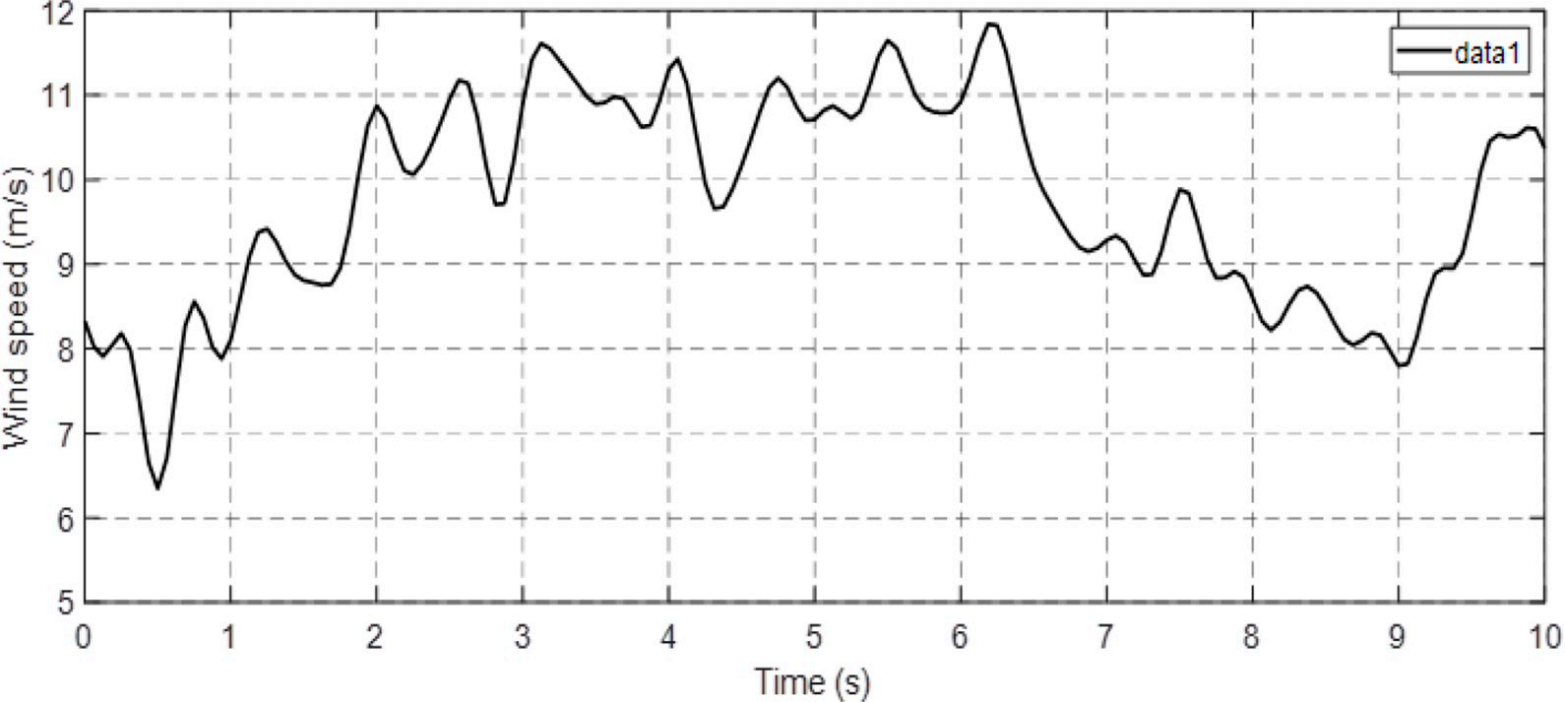
Wind profile of Al-Houceima Morocco city.

In order to evaluate the dynamic behavior and performance of the proposed novel system, simulations were conducted on a DFIG (1.5KW) using Matlab/Simulink software. The parameters of the DFIG utilized in this study can be found in the S1 Appendix. This section focuses on testing and comparing the performance of Classical-DPC and Artificial Neural Network-DPC approaches with reference values. To this end, a wind profile extracted from the Moroccan city of Al-Houceima, as depicted in Fig 9, was employed and the simulation results are presented in the subsequent.

**Fig 10:**
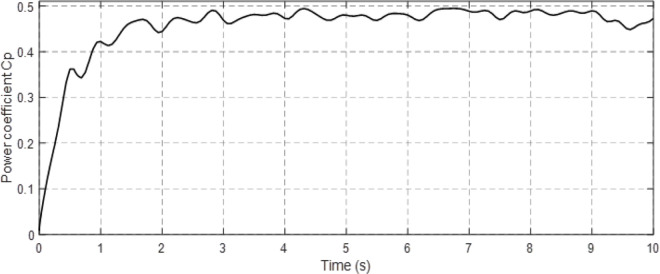
Power coefficient (*C*_*p*_).

**Fig 11:**
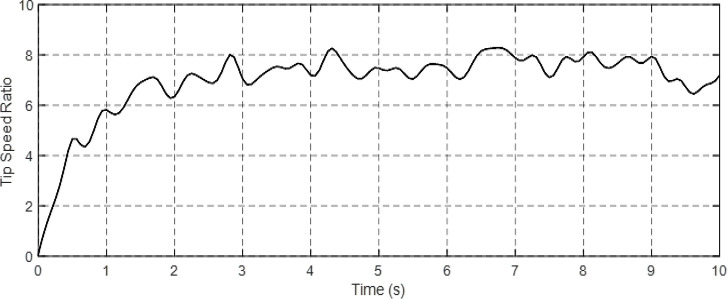
Tip speed ratio (λ).

**Fig 12:**
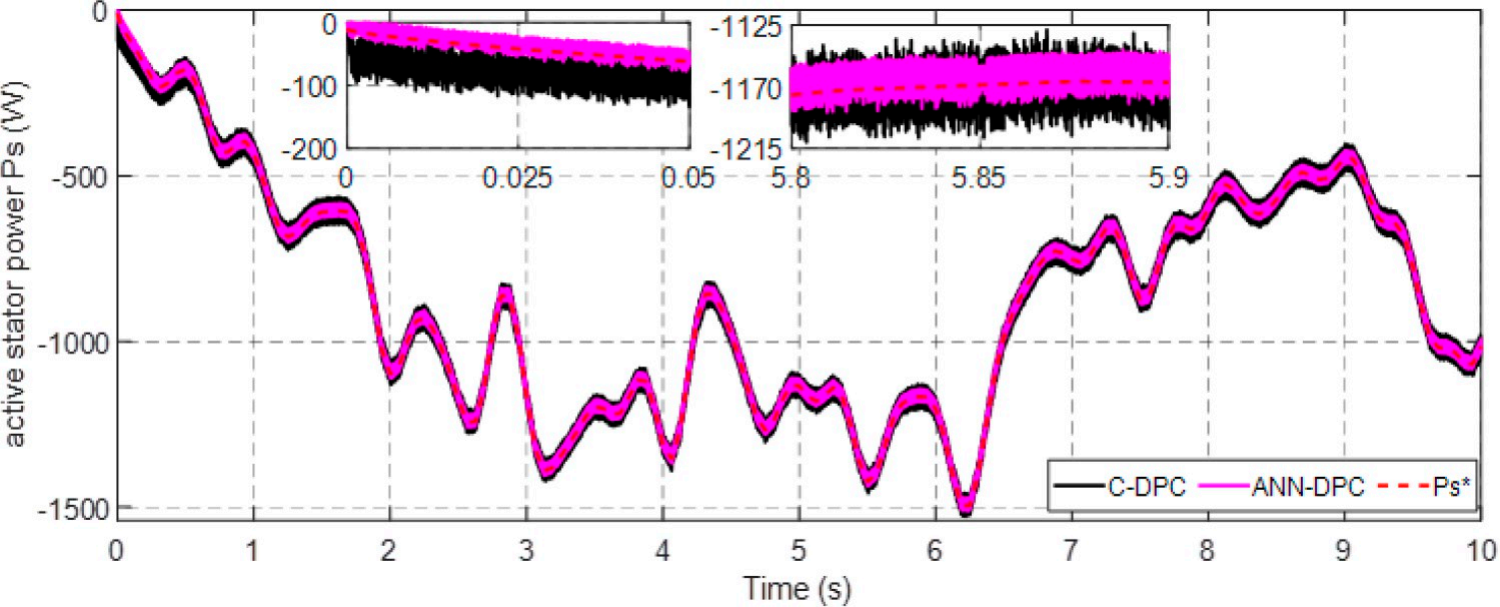
Active power.

**Fig 13:**
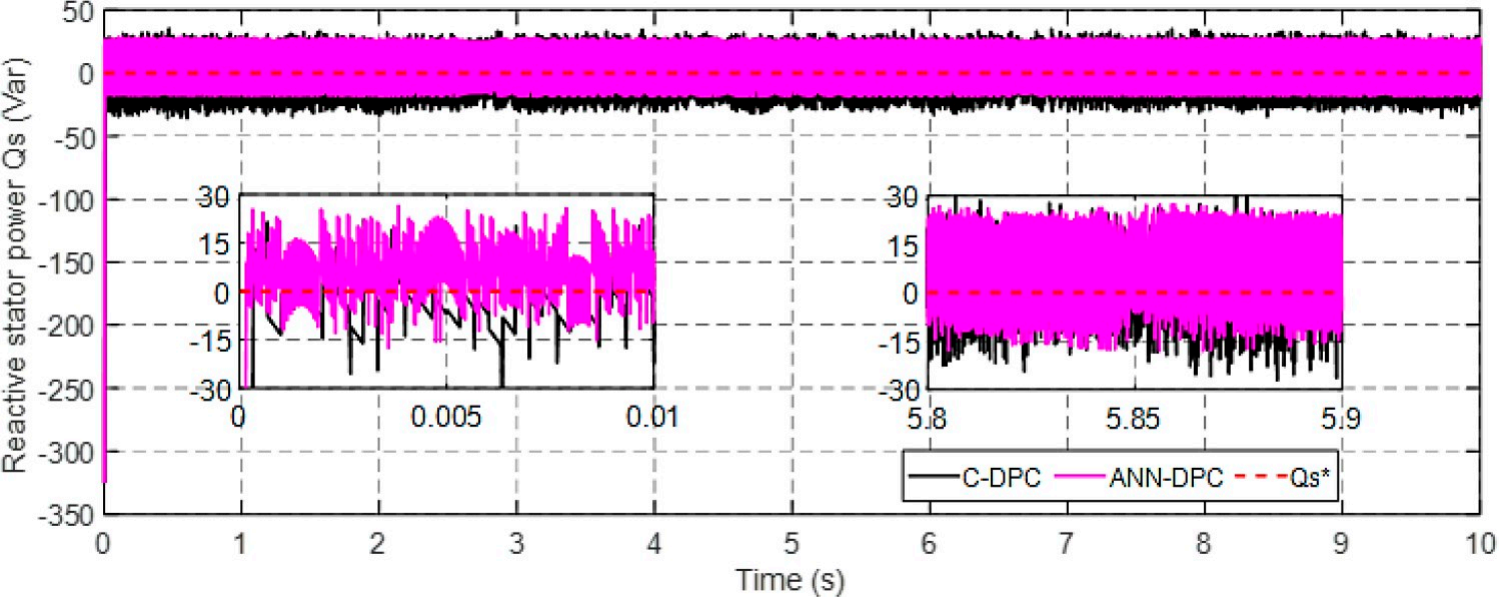
Reactive power.

**Fig 14:**
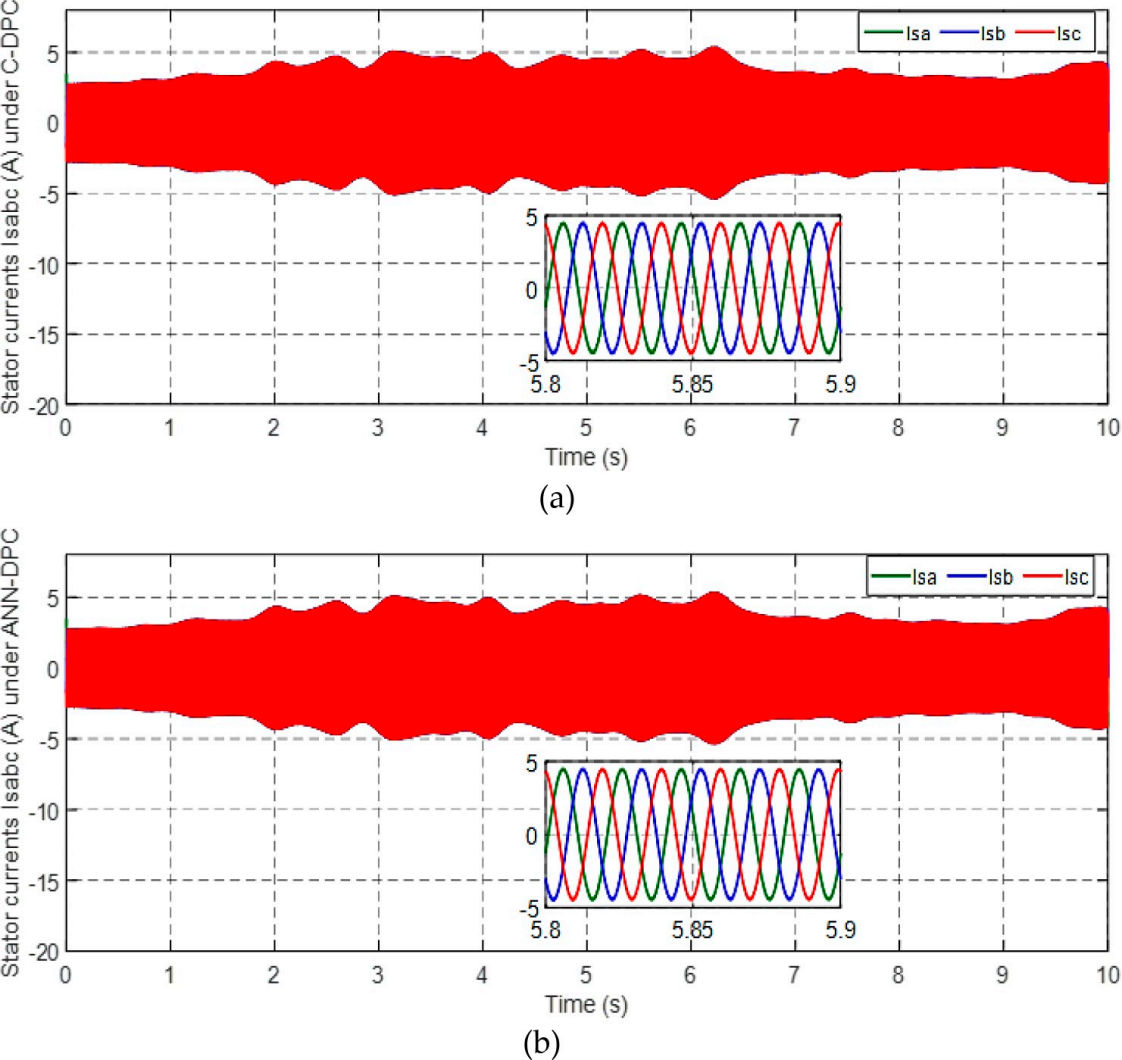
Stator current under C-DPC (a) and ANN-DPC (b).

**Fig 15:**
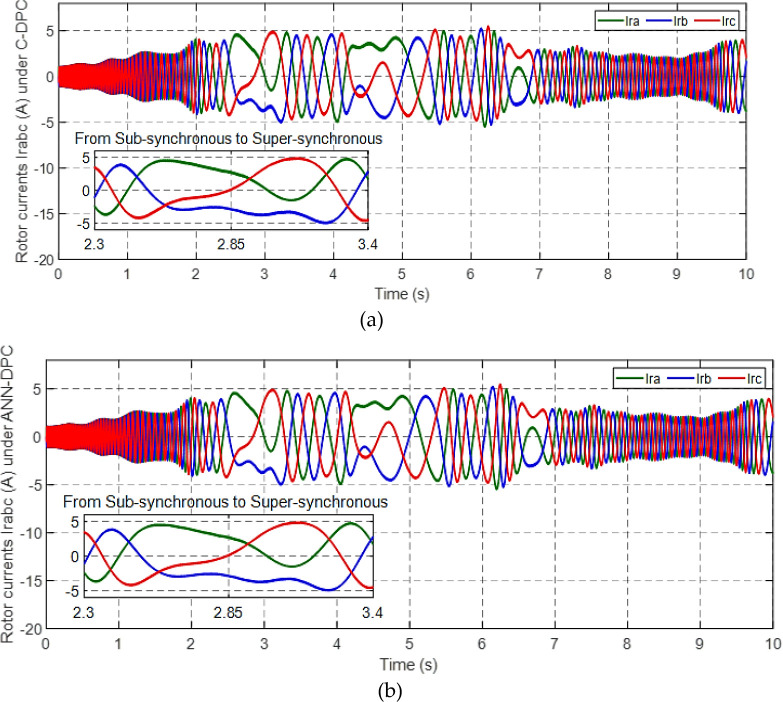
Rotor current under C-DPC (a) and ANN-DPC (b).

**Fig 16:**
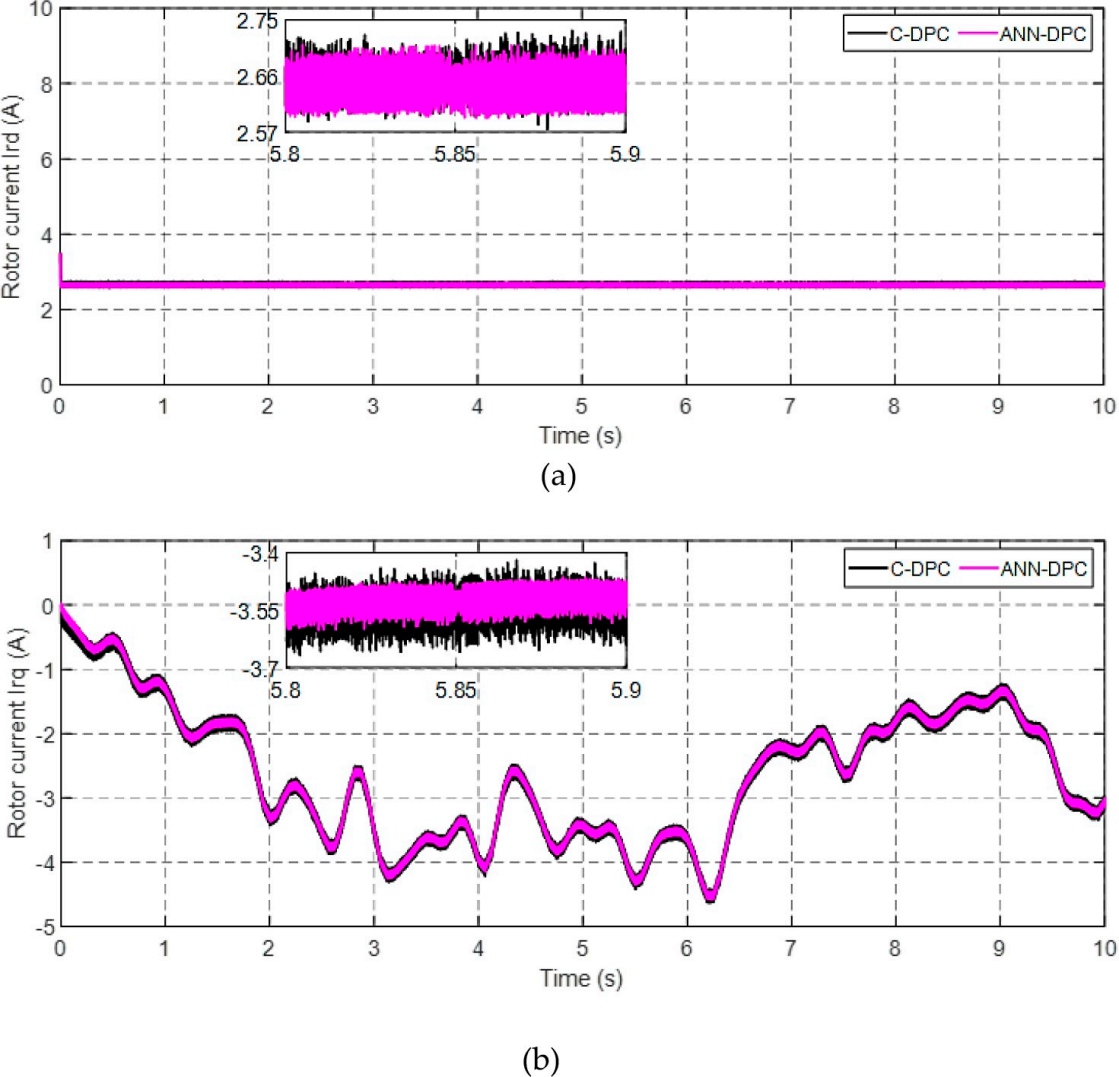
Direct (a) and quadrature (b) rotor currents.

**Fig 17:**
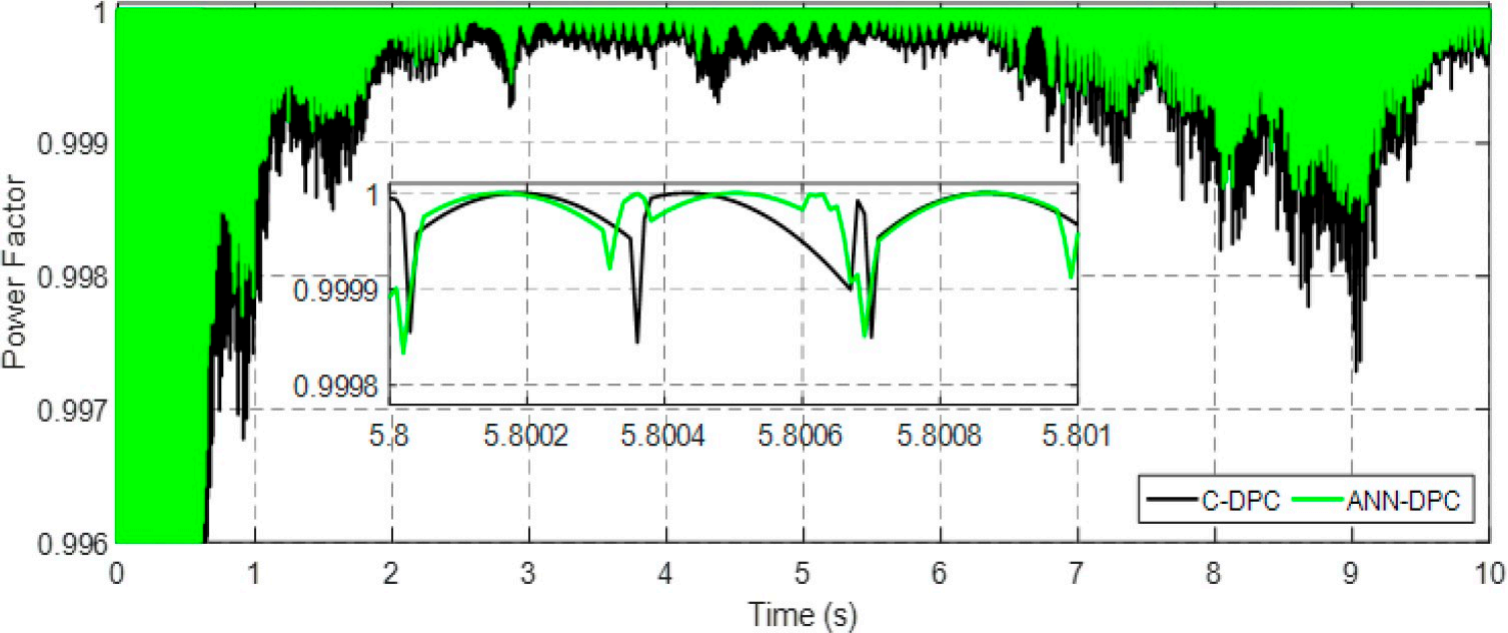
Power factor.

**Fig 18:**
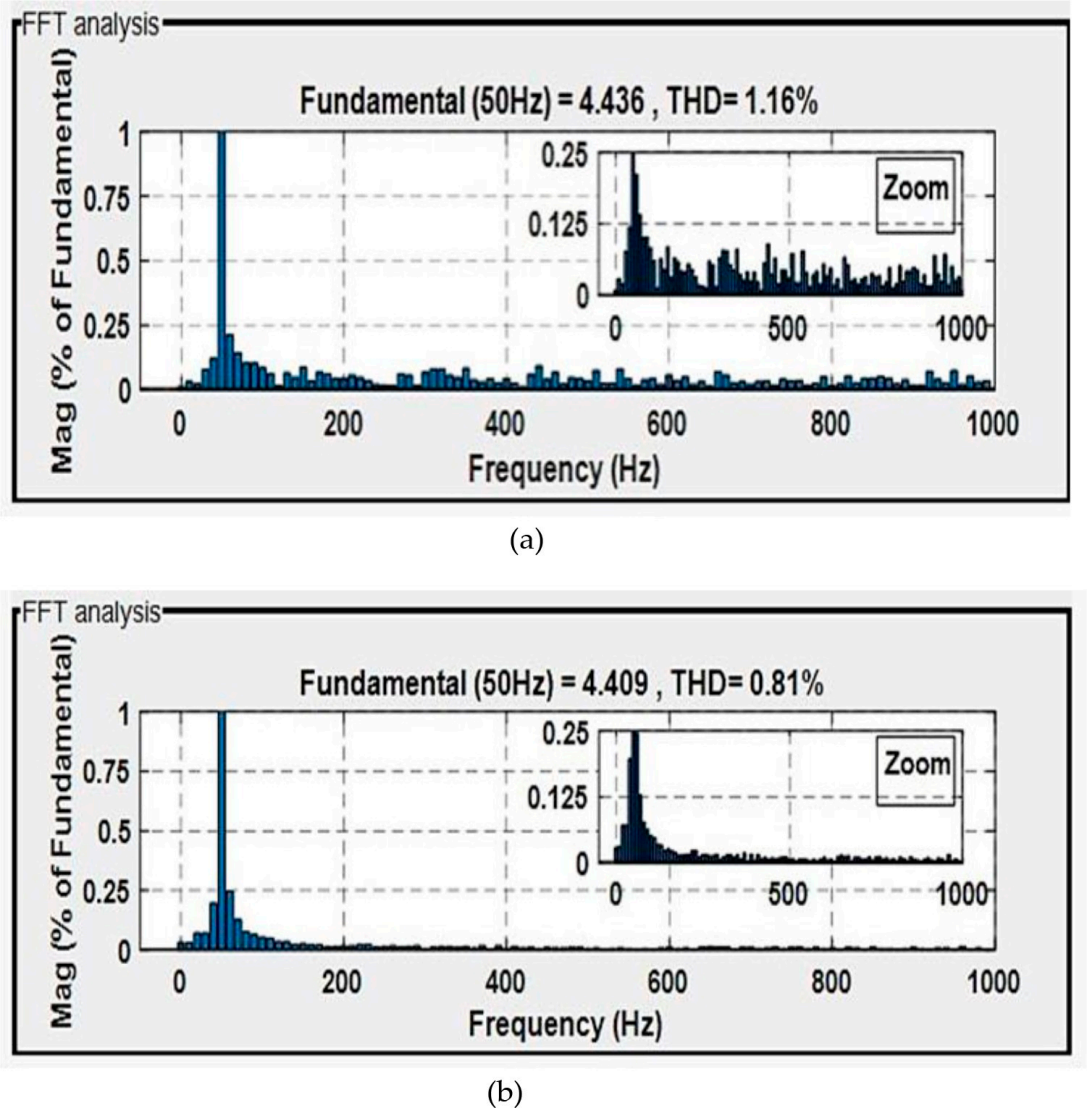
The total harmonic distortion of stator current by control: Classical-DPC (a) and ANN-DPC (b).

- Figs 10 and 11 depict the correlation between the power coefficient (Cp) and speed ratio (λ) with the wind profile. Changes in wind speed lead to variations in Cp and λ values. The Cp (λ, β) value is approximately 0.5, demonstrating variability, while the maximum tip speed ratio exhibits variability at approximately 8.1.
- Figs 12 and 13 illustrate the convergence of active and reactive power to their reference values, with Ps and Qs perfectly follow the reference. Even a slight change in wind speed can result in a significant variation in the extracted active power (ranging from 500W to 1500W), with Ps decreasing as wind speed decreases and vice versa. The reactive power reference remains fixed at zero to achieve a unity power factor on the network side.
- Figs 14 and 15 demonstrate the dependence of stator and rotor currents on the applied wind profile, both reaching a maximum value of 5A. It is observed that the value of the rotor/stator currents is linked to both the system and the reference value of Ps.
- Fig 16 presents the rotor current components in the (d, q) frame. The quadrature rotor current varies between approximately 1.2A and 4.7A, influenced by the active power. In contrast, the direct rotor current remains stable around 2.4A and is associated with reactive power.
- Fig 17 depicts the power factor of the system, which tends to be close to unity but exhibits slight ripples due to system operation and changes in wind speed.
- Fig 18 displays the analysis of harmonic distortion in the stator current drawn by the DFIG. The Total Harmonic Distortion (THD) resulting from the classical DPC is 1.16%, whereas the THD obtained by the intelligent DPC is 0.81%, signifying a significant reduction compared to that of the classical DPC approach.

## 6. Conclusion

This research has introduced a robust control method for Double-Fed Induction Generators (DFIG) using an Artificial Neural Network (ANN) in conjunction with an accurate wind profile. The primary objective of this work is to enhance the performance of Direct Power Control (DPC) in DFIG systems. The first section serves as an introduction to the paper. In the second section, the DFIG model is presented, along with the model of the associated wind turbine controlled by the Maximum Power Point Tracking (MPPT) strategy. Furthermore, stator field-oriented techniques are applied to simplify the DFIG model. The third section discusses the classical DPC approach, highlighting its advantages, drawbacks, and desired control objectives. The fourth section implements an intelligent DPC approach that combines the Pulse Width Modulation (PWM) strategy with a Multilayer Perceptron Artificial Neural Network (MLP-ANN). This intelligent approach enables the control of the stator powers of the DFIG. The fifth section is of utmost importance as it showcases the simulation results of the two controllers using MATLAB/SIMULINK. The main findings of this study can be summarized as follows:

- The DPC maintains its robustness and fast response characteristics.
- The ANN-DPC exhibits superior performance when dealing with varying wind speed profiles.
- The proposed control approach successfully reduces the total harmonic distortion (THD) of the current and mitigates power ripples.
- The efficiency of wind power conversion and the power factor in the system validate the effectiveness of the intelligent DPC technique employed in this study.

One potential direction for future research based on the finding of this paper could involve the investigation and implementation of advanced machine learning algorithms, such as deep learning techniques, to further enhance the control and performance of double-fed induction generators These advanced algorithms have the potential to learn and adapt to complex wind profiles, optimize control strategies, and improve the overall efficiency and reliability of DFIG systems. Additionally, exploring the integration of advanced optimization techniques, such as genetic algorithms or particle swarm optimization, with the intelligent control approach could offer opportunities for further improvements in the system’s performance and stability.

## Supporting information

S1 Data (RAR)

S1 Appendix (DOCX)

## Abbreviations

β (Degree °): Blade pitch angle
λ: Tip Speed Ratio
Cp: Power coefficient
ρ (Kg/m3): Air density
V (m/s): Wind speed
R (m): Blade radius
Ω_g_ (rad/s): Mechanical speed in the generator side
P_t_ (W): Wind turbine power
p: Number of pole pairs
V_dc_: DC-link voltage
P_s_(W), Q_s_ (Var): Stator active and reactive powers
ϕ_s_, ϕ_r_ (Wb): Stator and rotor fluxes
V_s_,V_r_ (V): Stator and Rotor voltages
i_s_, i_r_ (A): Stator and rotor currents
ω_s_, ω_r_ (rad/s): Stator and rotor pulsations
ANN: Artificial Neural Network
RSC: Rotor side Converter
GSC: Grid side Converter
