# Supplementary material for: Enhancing the control of doubly fed induction generators using artificial neural networks in the presence of real wind profiles
Source: PLoS One. 2024 Apr 17;19(4):e0300527. doi: 10.1371/journal.pone.0300527 (PMC11023233; doi:10.1371/journal.pone.0300527)
Supplement: S1 Appendix — (DOCX) [file pone.0300527.s002.docx]

**S1 Appendix**

**Table2. Parameters of the ANN**

| ANN parameters | | Input layer | Output layer | Proposed structure | Number of repetitions | Neural network | MLP training process | Activation functions | Adaption learning function |  |
| --- | --- | --- | --- | --- | --- | --- | --- | --- | --- | --- |
| Value/  Methods | *ANNC-P_s_* | ** | *v_dr-ref_* | 2-5-5-5-1 | 100 | MLP network | Levenberg Marquardt algorithm | Tansig | Trainlm |  |
|  | *ANNC-Q_s_* | ** | *v_qr-ref_* |  |  |  |  |  |  |  |

**Table3. Parameters of the DFIG**

| Rated power | *P_n_ = 1.5KW* |
| --- | --- |
| Stator rated voltage | *v_s_=220/380 V* |
| Stator current | *i_sn_=5.2A* |
| Rotor current | *i_rn_=8.5A* |
| Number of pair of poles | *p=2* |
| Stator rated frequency | f_s_=50Hz |
| Stator resistance | *R_s_ =1.18Ω* |
| Stator resistance | *R_r_ =1.66Ω* |
| Stator inductance | *L_ss_=0.20H* |
| Rotor inductance | *L_rr_=0.18H* |
| Mutual inductance | *L_m_=0.17H* |
| Rotor radius | *R=1m* |
| Number of blades | *3* |
| Gearbox gain | G=2 |
| Friction coefficient | *f=0.0027 N.m.s/rad* |
| Moment of inertia | *J=0.04kg.m^2^* |
